# Millimeter-wave channel modeling in a VANETs using coding techniques

**DOI:** 10.7717/peerj-cs.1374

**Published:** 2023-05-31

**Authors:** Arshee Ahmed, Haroon Rasheed, Ali Kashif Bashir, Marwan Omar

**Affiliations:** 1 Department of Electrical Engineering, Bahria University, Karachi, Sindh, Pakistan; 2 School of Business, Woxsen University, India, India; 3 Department of Computing and Mathematics, Manchester Metropolitan University, Manchester, UK; 4 Information Technology and Management, Illinois Institute of Technology, Chicago, United States

**Keywords:** Bit error rate, Space-time block code, Reed Solomon, Wireless communication, Ultra-reliability, PER, Beamforming, Doppler effecf, Throughput, Coding

## Abstract

The Vehicular ad-Hoc Network (VANET) is envisioned to ensure wireless transmission with ultra-high reliability. In the presence of fading and mobility of vehicles, error-free information between Vehicle to Vehicle (V2V) and Vehicle to Infrastructure (V2I) requires extensive investigation. The current literature lacks in designing an ultra-reliable comprehensive tractable model for VANET using millimeter wave. Ultra-reliable communication is needed to support autonomous vehicular communication. This article aims to provide a comprehensive tractable model for VANET over millimeter waves using Space-Time-Block-Coding (STBC) concatenated with Reed Solomon (RS) coding. The designed model provides the fastest way of designing and analyzing VANET networks on 60 GHz. By using the derived BER expressions and Reed Solomon coded doppler expression ultra-reliable vehicular networks can be build meeting the demands of massive growing volume of traffic. The performance of the model is compared with previous BER computational techniques and existing VANET communication systems, *i.e*., IEEE 802.11bd and 3rd generation partnership project vehicle to everything (3GPP V2X). The findings show that our proposed approach outperforms IEEE 802.11bd and the results are comparable with V2X NR. Packet Error Rate (PER), Packet Reception Ratio (PRR) and throughput are used as performance metrics. We have also evaluated the model on higher velocities of vehicles. Further, the simulation and numerical findings show that the proposed system surpass the existing BER results comprising of various modulation and coding techniques. The simulation results are verified by the numerical results there-by, showing the accuracy of our derived expressions.

## Introduction

In recent years, there has been significant demand for communication in Vehicular ad-Hoc Network (VANET) at high data rates. To meet the enormous communication demand in VANET, it is necessary to utilize the scarce resources, such as power and bandwidth, as effectively as feasible. Reliability, throughput, and accuracy are significant factors because of multipath fading and the rapid mobility of vehicle nodes. VANET communication system is susceptible to a certain level of noise, shadowing and fading. The Line of Sight (LOS) obstruction by vehicles results in an extra loss of the received power of approximately 10 dB (Abbas et al., 2015). In Va et al. (2016) atmospheric attenuation at 60 GHz was studied for number of vehicles, which is 15 dB/km and excessive path loss of 30 dB occurs for three obstructing vehicles. Analytical research on a Markov chain model is used in VANETs to examine the impact of channel fading on IEEE 802.11p. The performance is evaluated on Nakagami-m, Rayleigh, and Rician fading channels using BER (Shah et al., 2022). Highly mobile vehicular nodes and rapidly changing network topology throws many challenges in the dissemination of critical messages in VANET (Giripunje, Vidyarthi & Shandilya, 2022). Furthermore, the multipath propagation also leads to signal distortion and burst errors. Therefore, transmission reliability is very challenging in wireless channels. Forward error correction (FEC) is one of the most commonly used techniques to provide reliable communication.

The new Task Group TGbd was recently formed with the goal of exploring the future road map for vehicle to everything (V2X) and working towards a new standard known as next-generation V2X (NGV). 802.11bd is an 802.11p modification that specifies changes to the IEEE 802.11 medium access control (MAC) and physical layer (PHY) layers for V2X communications in the 5.9 and 60 GHz frequency bands. Currently, millimeter-wave (mmWave) massive Multi-Input Multi-Output (MIMO) is the most potential technology for vehicular communication (Yi & Zou, 2020). The mmWave signals are susceptible to high free-space path loss caused by high atmospheric attenuation (Shen et al., 2019).

Beamforming techniques are used in mmWave systems to mitigate the effects of large path losses by providing enough channel space (Kutty & Sen, 2015). The traditional MIMO system performance is enhanced due to multiple antennas systems at both links end. Thus providing an effective solution for future wireless communications systems as they provide high data rates. Beamforming signal processing techniques enable transmission and reception in the needed directions by applying appropriate antenna port phases and amplitudes by using what is known as the “weighting approach”. An adaptive algorithm instantly calculates the complex weights w_k_ to steer the null antenna pattern toward competing signals and the maximum antenna radiation pattern toward the chosen vehicular node.

In this research work, a VANET architecture is proposed using concatenated Space Time Block Coding-Reed Solomon (STBC-RS) coding in which the reliability of VANET communication is targeted. Error control coding technique using mmWave for minimizing errors is still a topic for rigorous investigation. Currently for ITS 5G communication, ultra-reliable networks are required meeting the demands of 1- 10^−5^ as indicated in Zrar Ghafoor et al. (2020). Moreover autonomous vehicles require reliable connectivity 1- 10^−7^ (Schulz et al., 2017).

Rayleigh fading channel is used in the proposed approach to model the fading characteristics of the channel. Because signal amplitude follow Rayleigh distribution when the line of sight (LOS) component gradually diminishes in vehicle to vehicle (V2V) communication. Rayleigh fading is most suitable in congested city roads, as concluded as a result of extensive simulations (Jameel et al., 2017).

### Contributions

The main contributions of this study are as follows:

- An ultra-reliable manipulable mathematical model is presented using RS coding along with 4 * 4 STBC. Transmit beamforming is employed by a complex weight expression of beamforming in the model.
- Two closed-form expressions for RS coding in the AWGN channel and in Rayleigh fading are derived. The numerical and simulation results have been obtained, which also verify our theoretical formulations.
- The results show that AWGN BER approximation outperforms conventional 64-QAM and M-PSK systems.
- The results also show that the proposed approach outmatched previous BER conputation approaches *i.e*., (Al-Barrak et al., 2017; Mergu, 2016; Tiwari, Hirwe & Dubey, 2013; Indoonundon & Pawan Fowdur, 2021; Saleh & Hasson, 2019; Hajiyat et al., 2019; Hamarsheh et al., 2022) shown in results and discussion section.
- The performance of the proposed model is compared with IEEE 802.11bd and V2X NR. The packet error probability (PEP), throughput and packet reception ratio (PRR) are used as performance metrics. It was observed that the proposed model outperforms IEEE 802.11bd. For designing V2X architectures RS error control coding performs better.
- MIMO-STBC along with RS can be used as a physical layer advancement technique in 802.11bd.
- We have also evaluated our results on higher velocities, achieving the BER of 10^−6^ to 10^−7^. Atlast the designed model is meeting the demands of ultra-reliability *i.e*., 1- 10^−5^ which is 0.99999.

## Related work

In Abuqamar, Hamamreh & Abewa (2021) STBC was combined with Orthogonal Frequency Division Multiplexing with Sub carrier-Power Modulation (OFDM-SPM) to analyze combination on a multi-path Rayleigh fading channel. BER and throughput were used as a performance metrics. It was concluded that using STBC the performance of original OFDM-SPM can be improved in terms of reliability. In Indoonundon & Pawan Fowdur (2021) to achieve ultra-reliable low latency communication in 5G, a detailed analysis of coding schemes was conducted. LDPC, polar codes, turbo codes were simulated using quadrature amplitude modulation (16QAM) and 64QAM. In this work, lower BER is obtained on increasing modulation order M in QAM modulation. RS coding was used in Cooperative-Intelligent Transport System (C-ITS) communications. The performance was analyzed and compared with Wyner–Ash codes. The result showed RS code outperformed with Wyner–Ash codes. Our designed model and derivations can be used in various C-ITS communications (Bocharova et al., 2019). RS performance was evaluated using STBC-MIMO systems concatenated with M-ary Quadrature Amplitude Modulation (M-QAM) and M-ary Phase-Shift Keying (MPSK) (Mane & Belsare, 2020). The performance of existing cooperative MIMO (C-MIMO) was improved using C-MIMO-STBC (Hai et al., 2021). It has been proved that wireless link quality can be improved by MIMO STBC while keeping transmitted power or frequency bandwidth constant. The performance of IEEE 802.11p was improved by implementing Multiple Input Single-Output (MISO) with Orthogonal Frequency-Division Multiplexing (OFDM) system. The authors analyzed the impact of time-varying channel on the performance of Alamouti STBC in OFDM systems (Youssefi & Mouhsen, 2020). 802.11p could not offer multi antenna communication. STBC-OFDM was used to analyze the performance of multi antennas. We have used STBC-RS channel model for improving reliability in VANET communication. In Triwinarko, Dayoub & Cherkaoui (2021) LDPC along with MIMO-STBC was used to improve the physical layer of 802.11p. STBC was combined with orthogonal frequency division multiplexing with OFDM-SPM to analyze combination on a multi-path Rayleigh fading channel. BER and throughput were used as a performance metrics. It was concluded that using STBC the performance of original OFDM-SPM can be improved in terms of reliability.

RS code was combined with Spatial Modulation (SM) and STBC along with cooperated source and relay (Zhao et al., 2022). Two closed-form approximations for BER Bose–Chaudhuri–Hocquenghem (BCH) coding and Alamouti Space Time Block Coding (BCH-ASTBC) were proposed for millimeter-wave VANET communication. The performance of conventional ASTBC equation was improved and the results were compared on different code rates (Ahmed, Rasheed & Liyanage, 2021).

The performance of WDM using M-ary DPPM schemes was enhanced under Amplified Spontaneous Emission (ASE) noise effects, InterChannel Crosstalk (ICC), and Atmospheric Turbulence (AT) (Yousif, Elsayed & Alzalabani, 2019). Due to multi-path fading and vehicle mobility, VANET communication is difficult in terms of reliability and throughput. The high mobility of vehicular communications, randomness in channel dynamics, and link interference are all significant challenges for vehicular networking. Many approaches have been investigated in this context to improve the efficiency of V2V and V2I communication. Researchers have expressed interest in using cooperative communications within vehicular networks to mitigate the impact of these challenges and improve reliability by allowing nodes to collaborate (Ahmed & Gharavi, 2018). Spatial multiplexing MIMO was employed by modifying the existing IEEE 802.11p standard for high throughput. Spatial multiplexing MIMO supports real-time data transfer in a non-line-of-sight environment (Dey, Akl & Chataut, 2020).

Cochannel interference degrades the system performance of mmWave radars mounted on vehicles. VANET-assisted interference mitigation approach was proposed that enables vehicles to coordinate their spectrum usage *via* multiple access. The proposed scheme was tested *via* a case study to show that the proposed scheme is useful in dense traffic with high mobility of vehicles (Zhang et al., 2020). MIMO systems are reliable and have high capacity. A beamformer transceiver design was proposed for the MIMO orthogonal frequency-division multiplexing (OFDM) system. It was used for highly reliable and spectrally efficient operation under the most damaging jamming attacks (Jagannath, Jagannath & Drozd, 2020).

In Ali et al. (2019) an architecture using massive MIMO was proposed. In the proposed architecture, vehicles are controlled using Global System Mobile (GSM) towers. GSM towers are equipped with 5G technology where towers can send and receive real-time information like the weather forecast, current temperature and city situation that comes under tower’s control.

The diversity gain was obtained by the STBC in wireless communication. The basic idea of exploiting transmit diversity was first developed by Tarokh, Jafarkhani & Calderbank (1999) and then a simpler structure was later introduced by Alamouti (1998). Later on, Almouti’s work was extended and developed to originate space time block codes.

Full duplex Non-Orthogonal Multiple Access (NOMA)-MIMO was employed in V2X systems and closed-form approximations for ergodic capacity were derived (Zhang et al., 2019). The transmission of multiple copies of data stream *via* the number of antennas can be achieved using space-time block codes MIMO. MIMO systems are used to improve data-transfer reliability by utilizing the various received data versions. The decoding algorithm is simple in nature and accomplishes full diversity specified by the number of transmit antennas at a radio receiver. STBC are complex and have same key features like Alamouti (1998). Further, their encoding and decoding schemes are also similar (Santumon & Sujatha, 2012). The data stream is first encoded in blocks when using STBC. These data blocks are then distributed across multiple antennas that are spaced apart. In Ehsanfar et al. (2022), the performance of three different frame structures of IEEE 802.11p, IEEE 802.11bd-draft, and a Unique-Word (UW)-based physical layer (PHY) were compared for vehicle-to-everything communication. Based on simulation results, it was determined that the UW-based PHY achieved interference-free channel estimation performance using a low complexity technique.

IEEE 802.11p does not perform well for high mobility networks. It uses Distributed Coordination Function (DCF) for communication between wireless nodes. In Hussain et al. (2017), a RSU-based efficient channel access scheme was proposed for VANETs under high traffic and mobility.

## Proposed model

**Figure 1:**
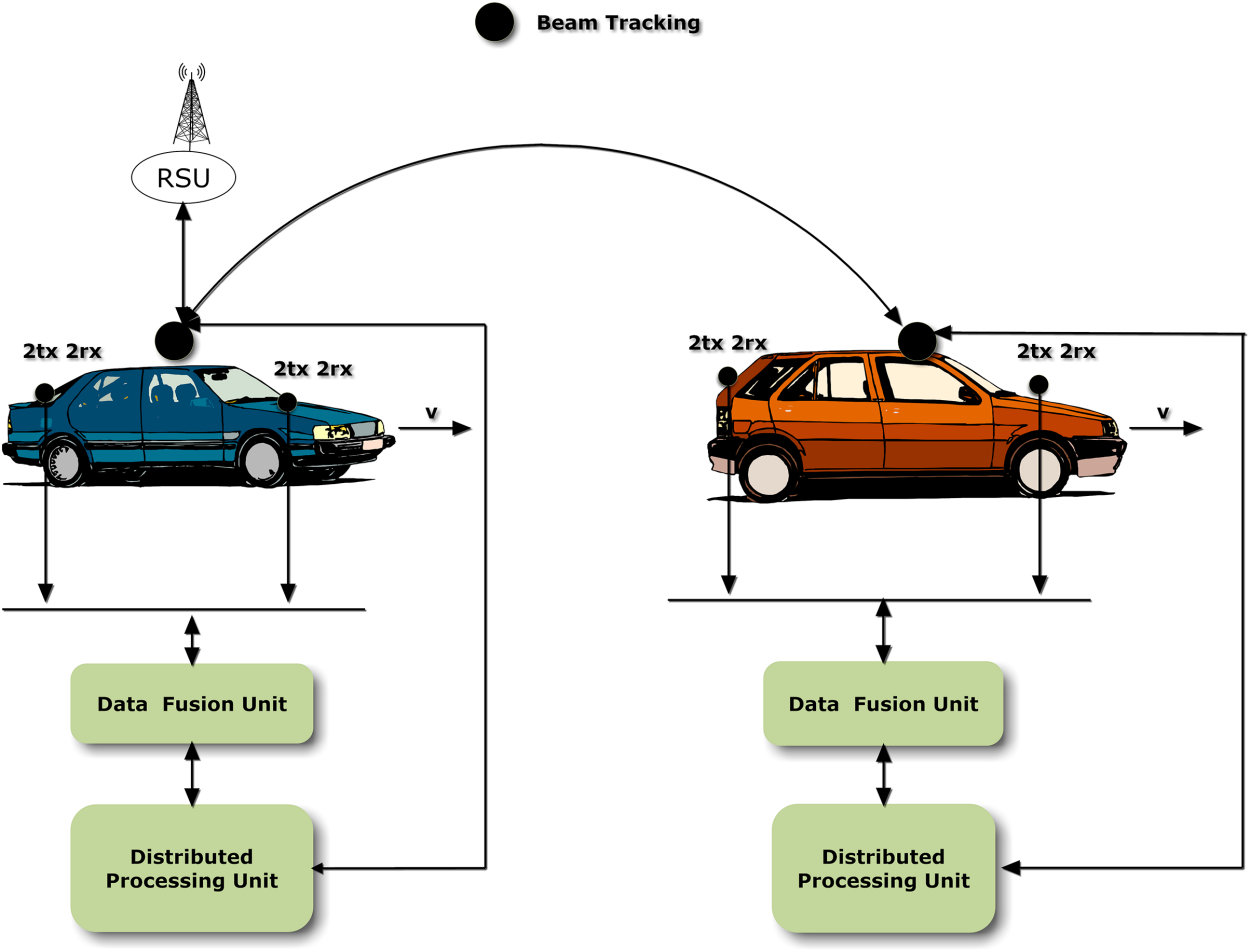
System model for RS-STBC.

A system model for VANET communication is described in Fig. 1 in which V2V and vehicle-to- road side unit (V2RSU) communication occurs. The sensors placed on vehicles receive information *via* four receiving antennas. The received information is passed to the data fusion unit which comprises of RS and STBC, where the received information *via* multiple antennas is fused. The information is sent to distributed processing unit, where the data is processed and then it is transferred to other vehicle.

**Figure 2:**
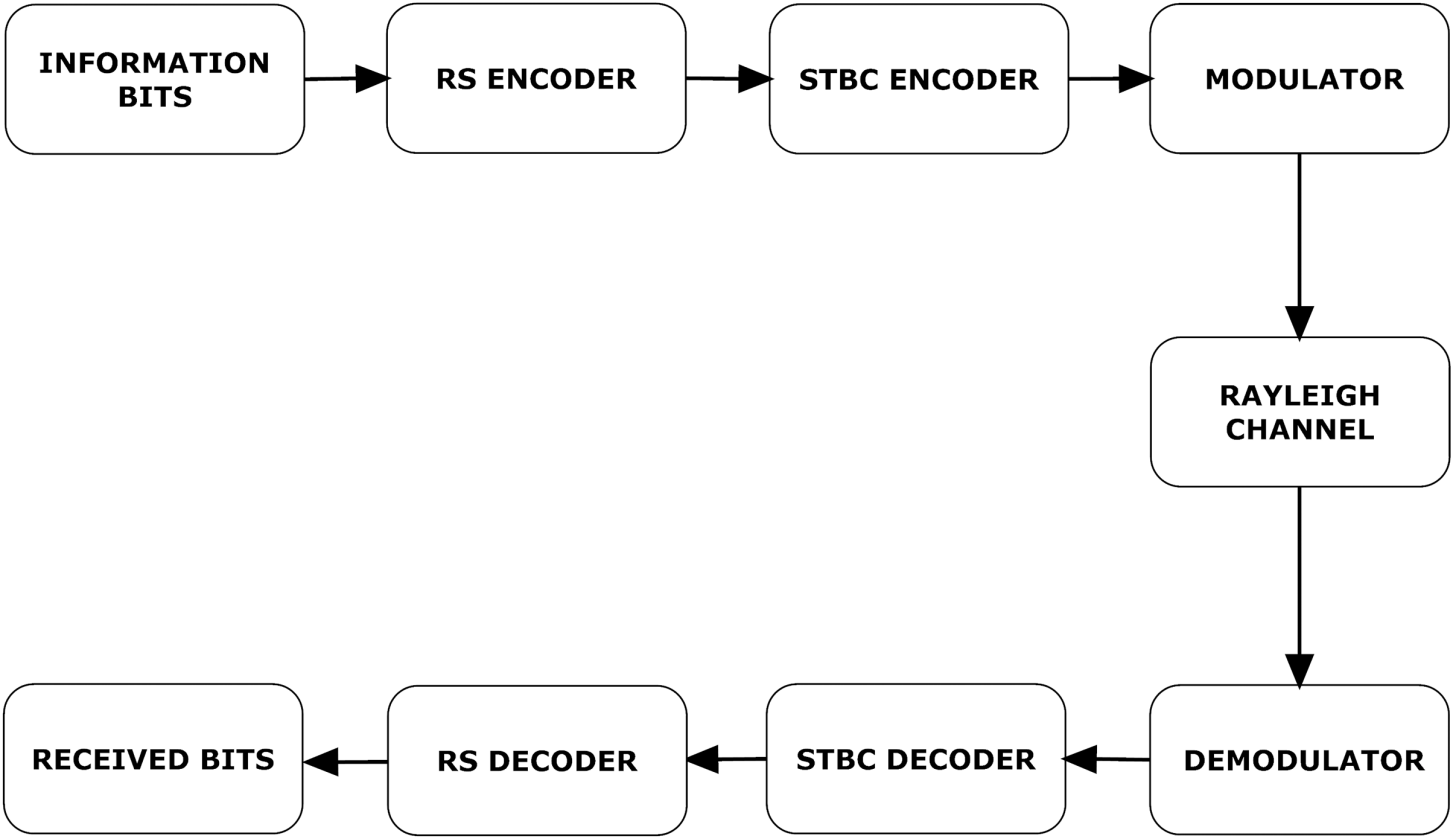
VANET communication process.

A detailed communication process is illustrated in Fig. 2. Information bits are encoded with RS encoder. Let i(x) denote information bits and g(x) denote generator polynomial. RS encoded bits can be written as,

$c(x) = i(x)g(x)$

where c(x) represents RS encoded message bits. Now c(x) is transmitted *via* four transmitting antennas using STBC coding. These signals are modulated and transmitted through a channel.

**Figure 3:**
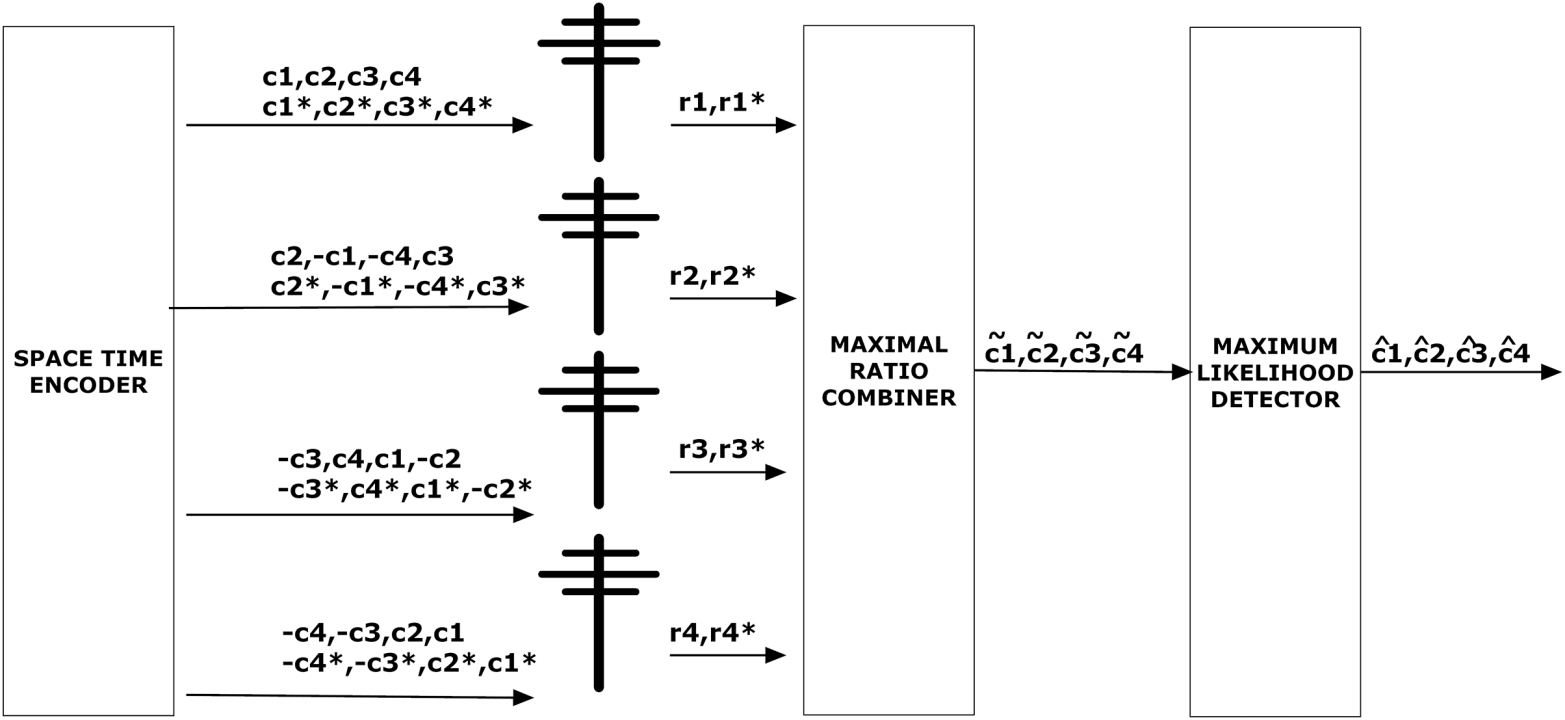
STBC encoder and decoder.

Figure 3 depicts the STBC encoder and STBC decoder. STBC decoder comprises of maximal ratio combining (MRC) and maximum likelihood (ML) respectively. Since there are four receivers in the system model, each receiver will receive either 0 or 1. The received signals can be represented as,

$\left[ {\matrix{ {{r_1}} \cr  {{r_2}} \cr  {{r_3}} \cr  {{r_4}} \cr  {{r_5}} \cr  {{r_6}} \cr  {{r_7}} \cr  {{r_8}} \cr  } } \right] = \left[ {\matrix{ {{c_1}} & {{c_2}} & {{c_3}} & {{c_4}} \cr  { - {c_2}} & {{c_1}} & { - {c_4}} & {{c_3}} \cr  { - {c_3}} & {{c_4}} & {{c_1}} & { - {c_2}} \cr  { - {c_4}} & { - {c_3}} & {{c_2}} & {{c_1}} \cr  {c_1^ * } & {c_2^ * } & {c_3^ * } & {c_4^ * } \cr  { - c_2^ * } & {c_1^ * } & { - c_4^ * } & {c_3^ * } \cr  { - c_3^ * } & {c_4^ * } & {c_1^ * } & { - c_2^ * } \cr  { - c_4^ * } & { - c_3^ * } & {c_2^ * } & {c_1^ * } \cr  } } \right] \times \left[ {\matrix{ {{h_1}} \cr  {{h_2}} \cr  {{h_3}} \cr  {{h_4}} \cr  } } \right] \times \left[ {\matrix{ {{w_1}} \cr  {{w_2}} \cr  {{w_3}} \cr  {{w_4}} \cr  } } \right]$

where w represents transmit beamforming weight vector *i.e*., w = [w1, w2, w3, w4]^T^ and [h1, h2, h3, h4]^T^ is channel response.

In the above matrix, signals transmitted and received by four antennas are mentioned in Fig. 3. The output of the MRC in Fig. 3 is explained in Eq. (4). Above matrics can be written as,

$\left[ {\matrix{ {{r_1}} \cr  {{r_2}} \cr  {{r_3}} \cr  {{r_4}} \cr  {{r_5}} \cr  {{r_6}} \cr  {{r_7}} \cr  {{r_8}} \cr  } } \right] = \left[ {\matrix{ {{h_1}} & {{h_2}} & {{h_3}} & {{h_4}} \cr  { - {h_2}} & {{h_1}} & { - {h_4}} & {{h_3}} \cr  { - {h_3}} & {{h_4}} & {{h_1}} & { - {h_2}} \cr  { - {h_4}} & { - {h_3}} & {{h_2}} & {{h_1}} \cr  {h_1^ * } & {h_2^ * } & {h_3^ * } & {h_4^ * } \cr  { - h_2^ * } & {h_1^ * } & { - h_4^ * } & {h_3^ * } \cr  { - h_3^ * } & {h_4^ * } & {h_1^ * } & { - h_2^ * } \cr  { - h_4^ * } & { - h_3^ * } & {h_2^ * } & {h_1^ * } \cr  } } \right] \times \left[ {\matrix{ {{c_1}} \cr  {{c_2}} \cr  {{c_3}} \cr  {{c_4}} \cr  } } \right] \times \left[ {\matrix{ {{w_1}} \cr  {{w_2}} \cr  {{w_3}} \cr  {{w_4}} \cr  } } \right]$

which can be represented as,

(1) $$\bar r = {H_{{\rm{ef}}}}c\bar w$$

In Eq. (1) H_ef_ is effective channel response.

Corresponding weights w_i_ can be described below where w_i_ represents array factor and amplitude weight are applied by w_b_ (Stepanets & Fokin, 2019),

(2) $${w_i} = \sum\limits_{b = 1}^N {{w_b}} {\exp ^{jN\psi }}$$

Equation (2) can be further simplified to normalized Eq. (3).

(3) $${{|}{w_i}{|}^2} = {1 \over N}{|}{{sin({{Nsin\psi } \over 2})} \over {sin{\psi \over 2}}}{|}$$

where N represents the transmitting antennas, and $\psi$ corresponds to the far-zone phase difference between adjacent elements, which can be calculated using the expression below (White & Reil, 2016).

**Figure 4:**
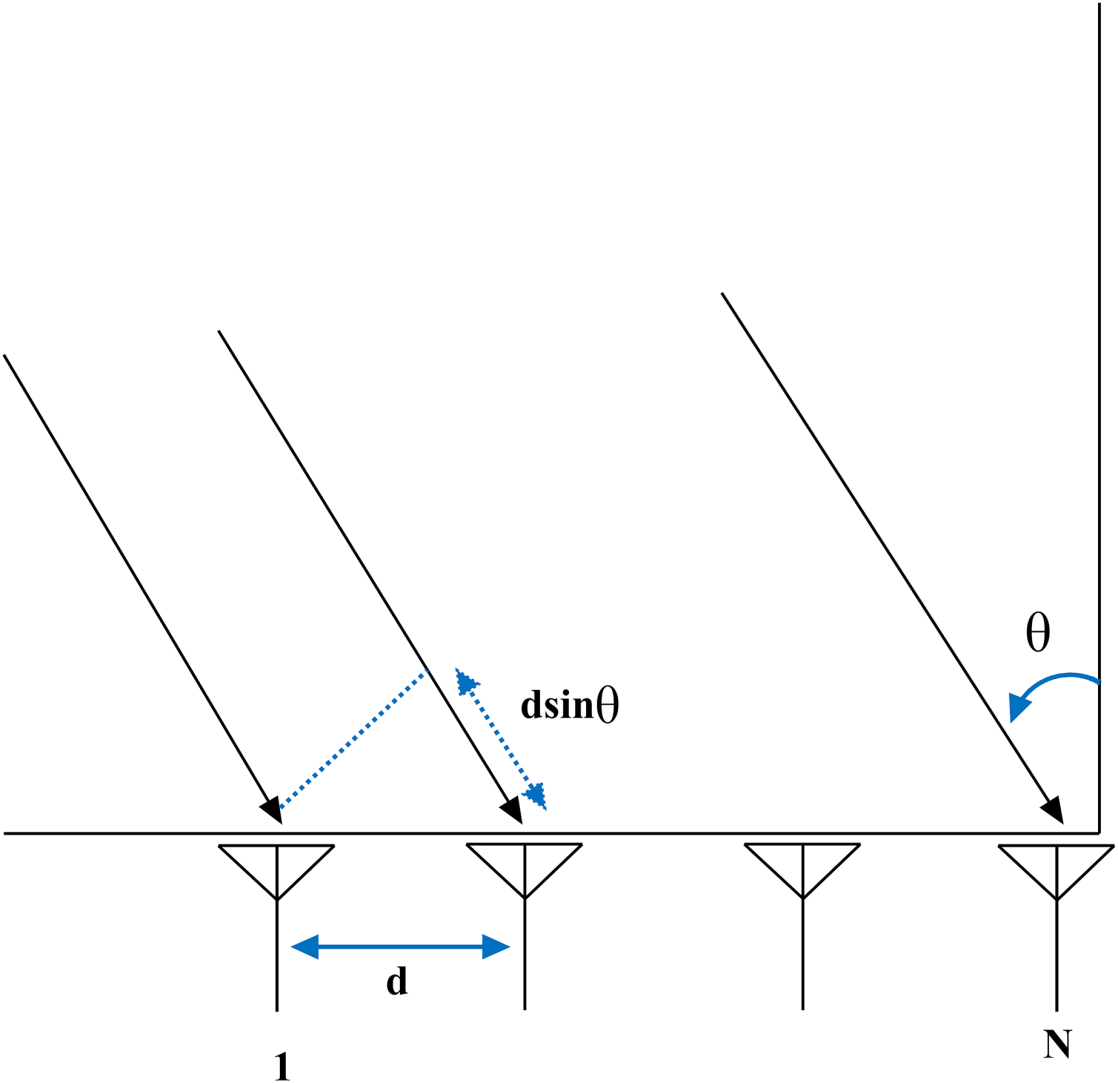
Beamforming in VANET.

$\psi = sdsin\phi sin\theta sin\phi + \Delta \psi$

where $\theta$ represents angle of arrival described in Table 1 and $\phi$ represents angle of reflection. s corresponds to periodicity of complex weight which is s = 2 $\pi$/ $\lambda$ and d represents equidistant spacing between elements N as described in Fig. 4. $\Delta \psi$ can be computed using beam steering formula as follows.

**Table 1:** Beamforming simulation parameters.

Parameter	Value
Frequency	60 GHz
$\theta$	$- {15^\circ }$ to ${15^\circ }$
*N*	2, 3, 4
$d$	30 mm, 40 mm, 50 mm

$\Delta \psi = {{2\pi dsin\theta } \over \lambda }$

The main lobe of an antenna array can be steered to a certain angle $\theta$ using phase offset $\Delta \psi$. $\phi$ is computed using expression $\phi$ = $\pi$sin $\theta$.

### Maximal ratio combining (MRC) at receiver end

In MRC, multiple receivers are used for signal reception. Equation (4) describes the relation between the signal arrived at MRC and transmitted by the MRC to ML (Andrei, 2012).

(4) $${\widetilde {c}} = H_{ef}^Hr$$

In Eq. (4) ${\widetilde {c}}$ is output of MRC (four output signals), r represents input of MRC and H_ef_^H^ corresponds to Hermitian matrix of effective channel response (Andrei, 2012).

Equation (4) can be written as,

$\left[ {\matrix{ {\widetilde{c_1}} \cr {\widetilde{c_2}} \cr {\widetilde{c_3}} \cr {\widetilde{c_4}} \cr } } \right] = \left[ {\matrix{ {h_1^ * } & {h_2^ * } & {h_3^ * } & {h_4^ * } & {{h_1}} & {{h_2}} & {{h_3}} & {{h_4}} \cr {h_2^ * } & { - h_1^ * } & { - h_4^ * } & {h_3^ * } & {{h_2}} & { - {h_1}} & { - {h_4}} & {{h_3}} \cr {h_3^ * } & {h_4^ * } & { - h_1^ * } & { - h_2^ * } & {{h_3}} & {{h_4}} & { - {h_1}} & { - {h_2}} \cr { - h_4^ * } & { - h_3^ * } & {h_2^ * } & { - h_1^ * } & { - {h_4}} & { - {h_3}} & {{h_2}} & { - {h_1}} \cr } } \right] \times \left[ {\matrix{ {{r_1}} \cr {{r_2}} \cr {{r_3}} \cr {{r_4}} \cr {r_1^ * } \cr {r_2^ * } \cr {r_3^ * } \cr {r_4^ * } \cr } } \right]$

The probability of error in MRC Rayleigh fading is described in Eq. (5) (Goldsmith, 2005: eq. (7.18)).

(5) $${p_e} = {\left({{1 - \Gamma } \over 2}\right)^L}\sum\limits_{l = 0}^{L - 1} {\left( \matrix{ L - 1 + l \cr l \cr} \right)} {\left({{1 + \Gamma } \over 2}\right)^l}$$

where L represents the number of the receiver. In Eq. (5), $\Gamma$ can be written as,

(6) $$\Gamma = \sqrt {{\gamma \over {1 + \gamma }}}$$

### Maximum likelihood (ML) estimator

After MRC, signals c(x) are sent to ML estimator as mentioned in Eq. (7). Afterward RS decoder receives signals and processes them.

(7) $$y(x) = c(x) + e(x)$$

The signal-to-noise ratio per receive antenna can be written as,

(8) $${\gamma _1} = {{{w_i}.m.{P_t}.N.{R_c}} \over {{\sigma ^2}}}$$

where P_t_ represents transmitted power in dB, m represents modulation index, $\sigma$ corresponds to standard deviation and R_c_ denotes code rate. SNR per symbol can be computed as,

(9) $${\gamma ^{STBC}} = {{\bar \gamma } \over {N{R_c}}}(\parallel H\parallel )_F^2$$

where $\parallel H\parallel$^2^_F_ represents Frobenius norm of matrix H.

### RS error probability in Rayleigh channel (Non line of sight model)

In this subsection, RS channel probability in Rayleigh fading is determined, *i.e*., non line of sight (NLOS) model. RS can detect and correct t = n − k/2 symbol errors where n represents the number of coded bits and k corresponds to the number of information bits. For detection and correction of errors d_min_ should be more than $\rm {n-k+1}$.

Probability of symbol error P_s_ is given as,

(10) $${P_s} = \sum\limits_{t + 1}^n {\left( \matrix{ n \cr j \cr} \right)} p_e^j{(1 - {p_e})^{n - j}}$$

where p_e_ is calculated using Eq. (4) and j = t + 1. The probability of error of RS in Rayleigh fading is written as,

(11) $${P_e} = \int_0^{ + \infty } \; {P_s}{1 \over {\bar \gamma }}ex{p^{ - {{{p_e}} \over {\bar \gamma }}}}d{p_e}$$

Substituting Eq. (10) into Eq. (11) gives,

(12) $${P_e} = \int_0^{ + \infty } {\sum\limits_{t + 1}^n {\left( \matrix{ n \cr j \cr} \right)} } p_e^j{(1 - {p_e})^{n - j}}d{p_e}.{1 \over {\bar \gamma }}ex{p^{ - {{{p_e}} \over {\bar \gamma }}}}$$

Using (Gradshteyn & Ryzhik, 2007: eq. (1.111), p.25)

(13) $${(a + x)^n} = \sum\limits_{t = 0}^n {\left( \matrix{ n \cr j \cr} \right)} {a^j}{x^{n - j}}$$

Expression (12) can be modified to,

(14) $${P_e} = \int_0^{ + \infty } {{{({p_e} + x)}^n}} {1 \over {\bar \gamma }}ex{p^{ - {{{p_e}} \over {\bar \gamma }}}}d{p_e}$$

Applying integration,

(15) $${P_e} = {1 \over {\bar \gamma }}ex{p^{ - {{{p_e}} \over {\bar \gamma }}}}\int_0^{ + \infty } {{{({p_e} + x)}^n}} d{p_e} - \int_0^{ + \infty } {\left({1 \over {\bar \gamma }}ex{p^{ - {{{p_e}} \over {\bar \gamma }}}}{d \over {d{p_e}}}\right)} \int_0^{ + \infty } {{{({p_e} + x)}^n}} d{p_e}$$

(16) $${P_e} = {1 \over {\bar \gamma }}ex{p^{ - {{{p_e}} \over {\bar \gamma }}}}.{{{{({p_e} + x)}^{n + 1}}} \over {n + 1}} - \int_0^{ + \infty } {\left({1 \over {\bar \gamma }}ex{p^{ - {{{p_e}} \over {\bar \gamma }}}}{d \over {d{p_e}}}\right)} {{{{({p_e} + x)}^{n + 1}}} \over {n + 1}}d{p_e}$$

Applying limits to p_e_.

(17) $${P_e} =  - {1 \over {\bar \gamma }}ex{p^{ - {{{p_e}} \over {\bar \gamma }}}}{{{{(x)}^{n + 1}}} \over {n + 1}} - \int_0^{ + \infty } {{\rm{ }}\left( {{1 \over {\bar \gamma }}ex{p^{ - {{{p_e}} \over {\bar \gamma }}}}{d \over {d{p_e}}}} \right)\left( { - {{{{(x)}^{n + 1}}} \over {n + 1}}} \right)d{p_e}} $$

Applying differentiation,

(18) $${P_e} = - {1 \over {\bar \gamma }}ex{p^{ - {{{p_e}} \over {\bar \gamma }}}}{{{{(x)}^{n + 1}}} \over {n + 1}} + \int_0^{ + \infty } {{1 \over {\bar \gamma }}} ex{p^{ - {{{p_e}} \over {\bar \gamma }}}}\left( - {1 \over {\bar \gamma }}\right){{{{(x)}^{n + 1}}} \over {n + 1}}d{p_e}$$

Applying integration finally we have,

(19) $${P_e} = - {1 \over {\bar \gamma }}ex{p^{ - {{{p_e}} \over {\bar \gamma }}}}{{{{(x)}^{n + 1}}} \over {n + 1}} - {1 \over {{{\bar \gamma }^2}}}{1 \over {{1 \over {\bar \gamma }}}}{{{{(x)}^{n + 1}}} \over {n + 1}}$$

The Eq. (19) represents closed-form approximation of BER of RS in Rayleigh fading.

### RS error probability in AWGN channel (Line of sight model)

BER of AWGN channel can be written as (Goldsmith, 2005: eq. (6.3)),

(20) $$BER = {{{p_e}} \over {lo{g_2}M}}$$

The BER of RS in AWGN channel can be written as,

(21) $${P_e} = \int_0^{ + \infty } {{{{p_e}} \over {lo{g_2}M}}} \sum\limits_{t + 1}^n {\left( \matrix{ n \cr j \cr} \right)} .p_e^j{(1 - {p_e})^{n - j}}d{p_e}$$

Using expression (14), we have,

(22) $${P_e} = \int_0^{ + \infty } {{{({p_e} + x)}^n}} {{{p_e}} \over {lo{g_2}M}}d{p_e}$$

After manipulating above expression,

(23) $${P_e} = {{{p_e}} \over {lo{g_2}M}}{{{{({p_e} + x)}^{n + 1}}} \over {n + 1}} - \int_0^{ + \infty } {\left({1 \over {lo{g_2}M}}{{{{({p_e} + x)}^{n + 1}}} \over {n + 1}}\right)} d{p_e}$$

After solving above expression,

(24) $${P_e} = - {{{p_e}} \over {lo{g_2}M}}{{{x^{n + 1}}} \over {n + 1}} + \int_0^{ + \infty } {{1 \over {lo{g_2}M}}} {{{x^{n + 1}}} \over {n + 1}}d{p_e}$$

After integration, second term becomes 0.

(25) $${P_e} = {{{p_e}} \over {lo{g_2}M}}{{{x^{n + 1}}} \over {n + 1}}$$

Equation (25) represents closed-form approximation of bit error probability of RS in AWGN channel.

### Doppler effect in proposed model

Consider the proposed model in Fig. 1 in which the vehicles are traveling with velocity v which is varying from 25 to 250 km/h. The closed-form expression of RS BER probability in Rayleigh fading *i.e*., Eq. (19) is mentioned below,

(26) $${P_e} = - {1 \over {\bar \gamma }}ex{p^{ - {{{p_e}} \over {\bar \gamma }}}}{{{{(x)}^{n + 1}}} \over {n + 1}} - {1 \over {{{\bar \gamma }^2}}}{1 \over {{1 \over {\bar \gamma }}}}{{{{(x)}^{n + 1}}} \over {n + 1}}$$

SNR under Doppler effect can be written as,

$\bar \gamma = {{{A^2}} \over {2*{\sigma ^2}}}.f\rm _{d}$

where A corresponds to magnitude of signal and f_d_ represents Doppler shift. Plugging value of $\bar \gamma$ in expression (26),

${P_e} = {{2.{\sigma ^2}} \over {{f_d}}}.ex{p^{{{{p_e}.2.\sigma } \over {{f_d}}}}}.{{{x^{n + 1}}} \over {n + 1}} + {{2.{\sigma ^2}} \over {{f_d}}}.{{{x^{n + 1}}} \over {n + 1}}$

Probability density function of f_d_ can be written as,

(27) $${P_{ed}} = \int_0^{ + \infty } {{1 \over \gamma }} .ex{p^{ - {{{f_d}} \over \gamma }}}.{P_e}d{f_d}$$

Substituting P_e_ in above expression,

(28) $${P_{ed}} = \int_0^{ + \infty } {\left({1 \over \gamma }.ex{p^{ - {{{f_d}} \over \gamma }}}\right)} .\left({{2.{\sigma ^2}} \over {{f_d}}}.ex{p^{{{{p_e}.2.\sigma } \over {{f_d}}}}}.{{{x^{n + 1}}} \over {n + 1}} +{{2.{\sigma ^2}} \over {{f_d}}}.{{{x^{n + 1}}} \over {n + 1}}\right)d{f_d}$$

(29) $${P_{ed}} = \int_0^{ + \infty } {{1 \over \gamma }} .ex{p^{ - {{{f_d}} \over \gamma } - {{{p_e}.2.{\sigma ^2}} \over {{f_d}}}}}.{{{x^{n + 1}}} \over {n + 1}}.{{2.{\sigma ^2}} \over {{f_d}}} + {1 \over \gamma }.ex{p^{ - {{{f_d}} \over \gamma }}}.{{2.{\sigma ^2}} \over {{f_d}}}.{{{x^{n + 1}}} \over {n + 1}}d{f_d}$$

Manipulating above expression,

(30) $${P_{ed}} = {1 \over \gamma }.{{{x^{n + 1}}} \over {n + 1}}\int_0^{ + \infty } e x{p^{ - {{{f_d}} \over \gamma } - {{{p_e}.2.{\sigma ^2}} \over {{f_d}}}}}.{{2.{\sigma ^2}} \over {{f_d}}} + \int_0^{ + \infty } e x{p^{ - {{{f_d}} \over \gamma }}}.{{2.{\sigma ^2}} \over {{f_d}}}$$

Applying integration by parts,

(31) $$\eqalign{
  & {P_{ed}} = {1 \over \gamma }.{{{x^{n + 1}}} \over {n + 1}}.({{2.{\sigma ^2}} \over {{f_d}}}.\int_0^{ + \infty } e x{p^{ - {{{f_d}} \over \gamma } - {{{p_e}.2.{\sigma ^2}} \over {{f_d}}}}} - \int_0^{ + \infty } {\left( {{{2.{\sigma ^2}} \over {{f_d}}}{d \over {df}}} \right)} \int_0^{ + \infty } e x{p^{ - {{{f_d}} \over \gamma } - {{{p_e}.2.{\sigma ^2}} \over {{f_d}}}}}  \cr 
  & \,\,\,\,\,\,\, + ex{p^{ - {{{f_d}} \over \gamma }}}\int_0^{ + \infty } {{{2.{\sigma ^2}} \over {{f_d}}}}  - \int_0^{ + \infty } e x{p^{ - {{{f_d}} \over \gamma }}}{d \over {df}}.\int_0^{ + \infty } {{{2.{\sigma ^2}} \over {{f_d}}}} )d{f_d} \cr} $$

In Gradshteyn & Ryzhik (2007): eq. (3.325), p.336,

$\int_0^{ + \infty } e x{p^{ - {{{f_d}} \over \gamma } - {{{p_e}.2.{\sigma ^2}} \over {{f_d}}}}} = {1 \over 2}.\sqrt {{{pi} \over {{1 \over \gamma }}}} .ex{p^{ - 2.\sqrt {{{2.{\sigma ^2}.{p_e}} \over \gamma }} }}$

Replacing above expression in expression (31) yields,

(32) $$\eqalign{
  & {P_{ed}} = {1 \over \gamma }.{{{x^{n + 1}}} \over {n + 1}}.({{2.{\sigma ^2}} \over {{f_d}}}.{1 \over 2}.\sqrt {{{pi} \over {{1 \over \gamma }}}} .ex{p^{ - 2.\sqrt {{{2.{\sigma ^2}.{p_e}} \over \gamma }} }} + \int_0^{ + \infty } {}   \cr 
  & \,\,\,\,\,\,\left( {{{2.{\sigma ^2}} \over {f_d^2}}} \right).{1 \over 2}.\sqrt {{{pi} \over {{1 \over \gamma }}}} .ex{p^{ - 2.\sqrt {{{2.{\sigma ^2}.{p_e}} \over \gamma }} }}  \cr 
  & \,\,\,\,\,\, + ex{p^{ - {{{f_d}} \over \gamma }}}\int_0^{ + \infty } {{{2.{\sigma ^2}} \over {{f_d}}}}  - \int_0^{ + \infty } e x{p^{ - {{{f_d}} \over \gamma }}}{d \over {df}}.\int_0^{ + \infty } {{{2.{\sigma ^2}} \over {{f_d}}}} ) \cr} $$

Applying integration and limits,

(33) $${P_{ed}} = {1 \over \gamma }.{{{x^{n + 1}}} \over {n + 1}}. \left({{2.{\sigma ^2}} \over {{f_d}}}.{1 \over 2}.\sqrt {{{pi} \over {{1 \over \gamma }}}} .ex{p^{ - 2.\sqrt {{{2.{\sigma ^2}.{p_e}} \over \gamma }} }} - ex{p^{{{{f_d}} \over {\bar \gamma }}}}.2.{\sigma ^2} + 2.{\sigma ^2}.{1 \over \gamma }.\int_0^{ + \infty } e x{p^{ - {{{f_d}} \over \gamma }}}\right)$$

Manipulating above expression yields to,

(34) $${P_{ed}} = {1 \over \gamma }.{{{x^{n + 1}}} \over {n + 1}}. \left({{2.{\sigma ^2}} \over {{f_d}}}.{1 \over 2}.\sqrt {{{pi} \over {{1 \over \gamma }}}} .ex{p^{ - 2.\sqrt {{{2.{\sigma ^2}.{p_e}} \over \gamma }} }}- ex{p^{ - {{{f_d}} \over {\bar \gamma }}}}.2.{\sigma ^2} + {1 \over \gamma }.{1 \over {{1 \over \gamma }}}.2.{\sigma ^2}\right)$$

Applying integration and limits,

(35) $${P_{ed}} = {1 \over \gamma }.{{{x^{n + 1}}} \over {n + 1}}.\left({{2.{\sigma ^2}} \over {{f_d}}}.{1 \over 2}.\sqrt {{{pi} \over {{1 \over \gamma }}}} .ex{p^{ - 2.\sqrt {{{2.{\sigma ^2}.{p_e}} \over \gamma }} }} - ex{p^{ - {{{f_d}} \over {\bar \gamma }}}}.2.{\sigma ^2} + 2*{\sigma ^2}\right)$$

Equation (35) represents RS coded Doppler shift expression in Rayleigh fading.

## Results and discussion

This section comprises of results and discussion of the proposed model. MatLab is used as a simulation platform.

In this research, LOS, NLOS, number of antenna elements N, equidistant spacing d (30 mm, 40 mm, 50 mm) and relative velocity v (25 km/h, 100 km/h, 250 km/h) are used as use cases.

**Figure 5:**
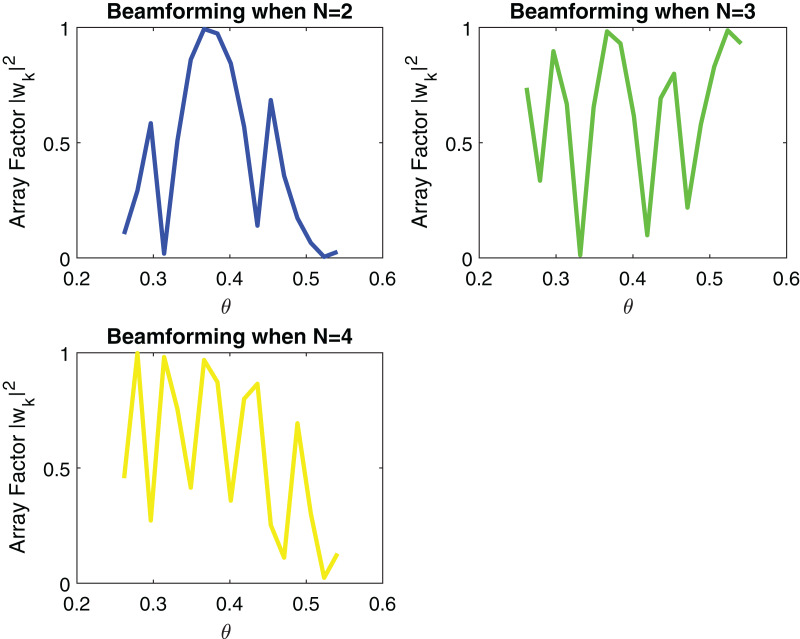
Signal received after Beamforming when number of elements are increasing.

**Figure 6:**
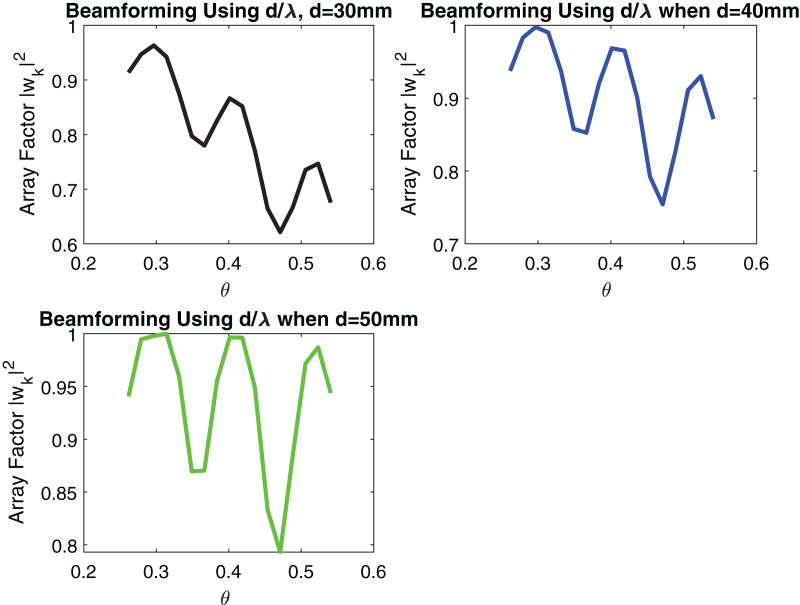
Signal received after Beamforming when distance between elements is increasing.

Figures 5 and 6 show the impact of the number of transmitting antennas and their equidistant spacing on directivity.

The simulation parameters are shown in Table 1.

From Fig. 5 it can be remarked that by increasing the number of transmitting antennas *i.e*., N which is varying from 2 to 4, directivity increases. When directivity increases, the beam gain increases and thus, the probability of signal loss reduces. d = 50 mm is used in simulation. However, the effect of increasing the number of transmitting antennas results in a larger number of side lobes as shown in Fig. 5. In beamforming, linear arrays with increasing equidistant element spacing will also produce grating lobes as described in Fig. 6. These grating lobes are unwanted energy that will be radiated to or received from undesired directions. If the equidistant spacing exceeds half a wavelength grating lobes start to appear in the visible region. To avoid this phenomenon following condition must be kept as,

$d\lt{\lambda \over 2}$

In case the distance between transmitting antennas exceeds one wavelength, the grating lobe levels start to equal the main lobe level. Since the array factor is periodic in nature, grating lobes will start to appear in the visible region coming from the invisible region. In this case, scan angle will be restricted. So the maximum value of scan angle must be,

$sin|\theta | = {\lambda \over d} - 1$

**Figure 7:**
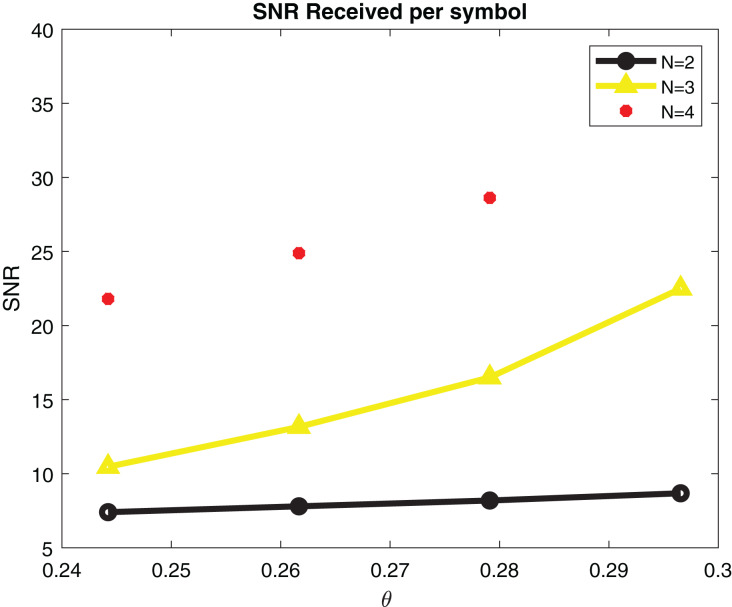
SNR per symbol in maximal likelihood detector.

**Figure 8:**
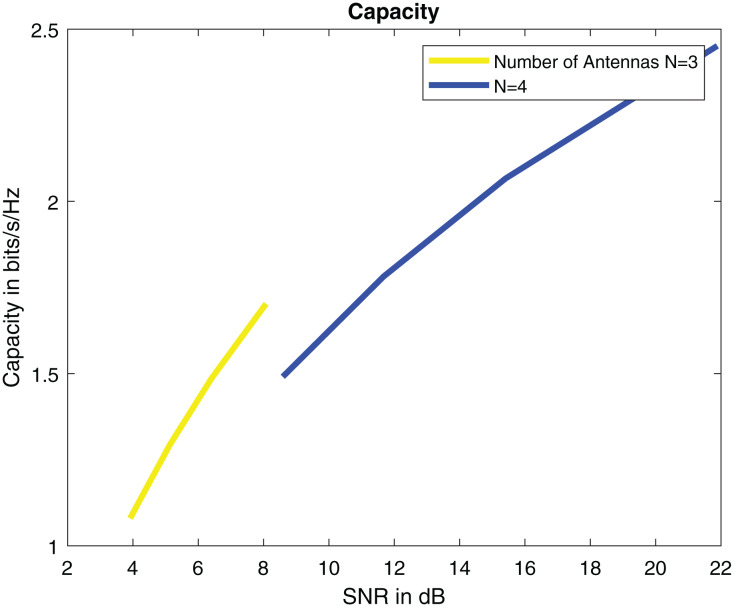
Capacity in bits/s/Hz.

For SNR calculation Eq. (8) is used. Simulation parameters are describe in Table 2. SNR per symbol is computed in a ML detector using expression (9). Figure 7 displays the graph of SNR per symbol *vs* angle of reflection theta. When increasing the angle of reflection, SNR per symbol also rises. By increasing the number of transmitters to N = 4, SNR also gets high. The capacity of a system is directly proportional to the number of transmitters. The capacity of our system is 2.5 bits/s/Hz when there are four transmitters as shown in Fig. 8.

**Table 2:** Simulation parameters.

Parameter	Value
No. of transmitting elements *N*	4
Transmit power ${P^t}$	10 dB
Modulation index $m$	1
Standard deviation ${\sigma ^2}$	9
Code rate ${R^c}$	0.9
Carrier frequency ${f^c}$	60 GHz
Data rate	7.31 Mbps
Packet size	100 Bytes

**Figure 9:**
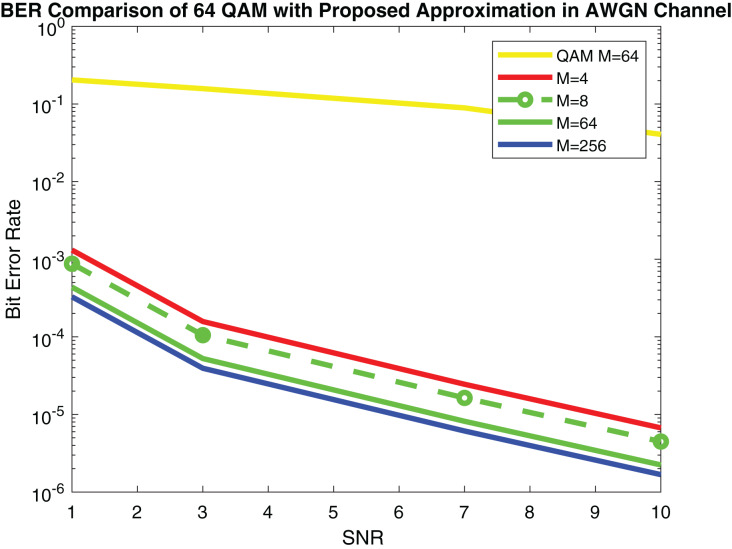
RS BER comparison of 64QAM with proposed approximation.

**Figure 10:**
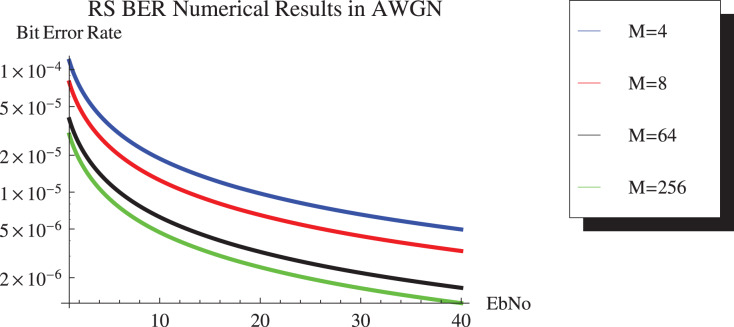
RS BER comparison of 64QAM with proposed approximation.

**Figure 11:**
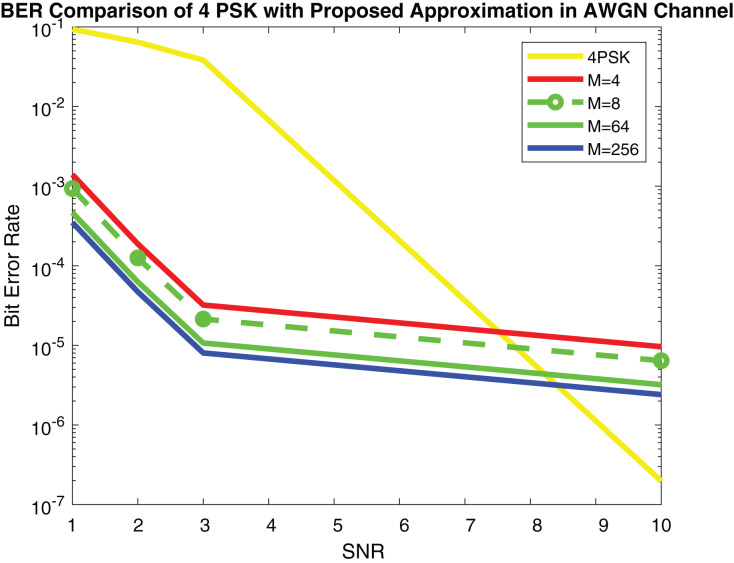
RS BER comparison of 4PSK with proposed approximation.

**Figure 12:**
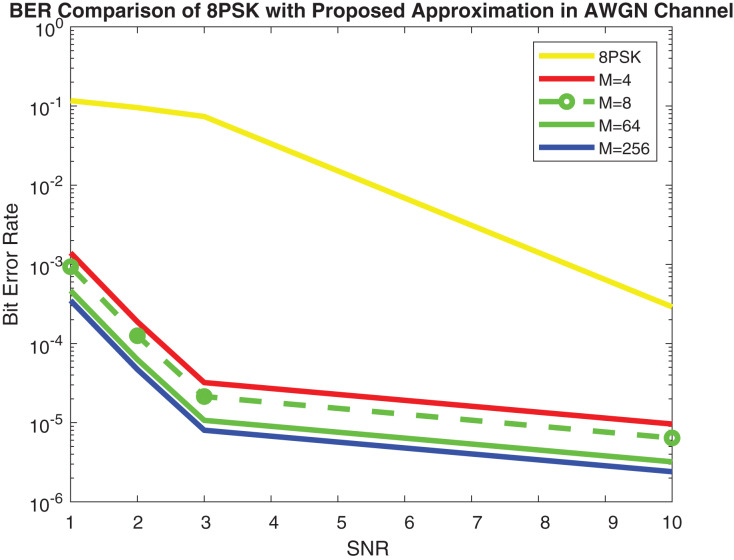
RS BER comparison of 8PSK with proposed approximation.

Figure 9 shows the simulation results and Fig. 10 depicts the numerical results of closed-form approximation of RS BER in the AWGN channel. It can be remarked from both figures that the results verify each other. On comparing our result with the traditional 64-QAM system it can figure out that the BER of 64-QAM is higher than our proposed approximation. The BER of the M-PSK is compared with our expression described in Figs. 11 and 12. The BER of M-PSK system is getting low on account of decreasing modulation order M. The BER of the 4PSK system is lower than the 8PSK system. However, the BER of proposed approximation gets low on account of increasing modulation order M. So our result outperforms both the 64-QAM and M-PSK system. The simulation parameter described in Table 2.

**Figure 13:**
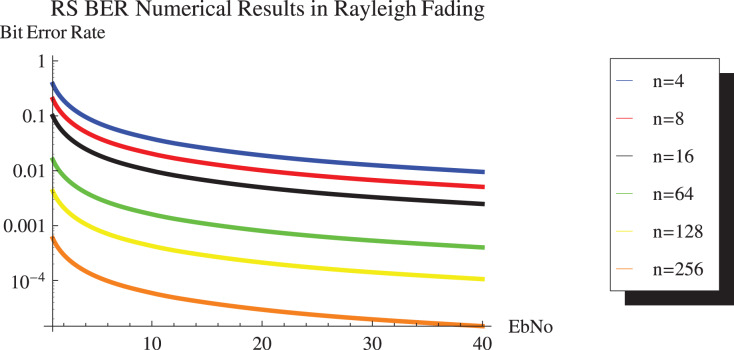
RS BER proposed approximation numerical results in Rayleigh channel.

**Figure 14:**
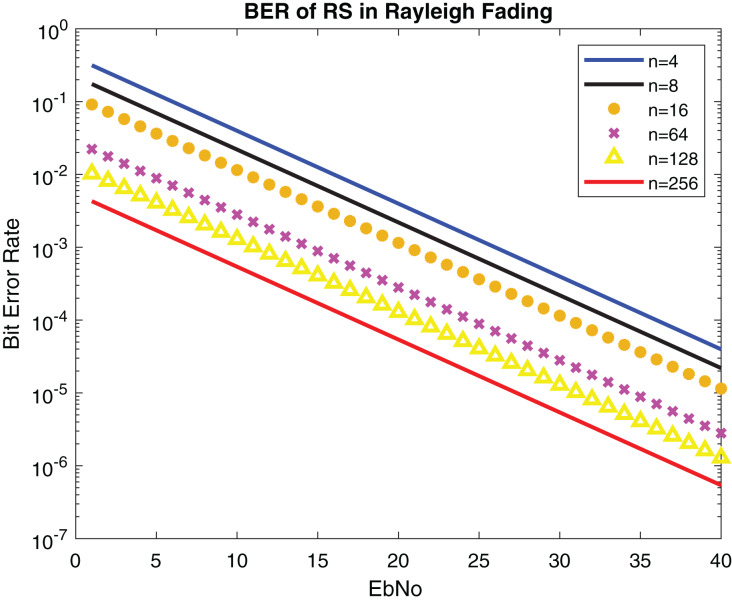
BER of RS in Rayleigh fading.

Figures 13 and 14 show the numerical results and simulation results of closed-form approximation of RS error probability in the Rayleigh channel. On comparing results based on n, it can be observed that error probability is getting depleted by increasing n.

Table 3 illustrates the comparative analysis of proposed model with previous studies in terms of BER. The results are analyzed using NLOS/LOS cases and modulation and coding types from different studies.

**Table 3:** Comparative analysis of proposed model with previous studies in terms of BER.

Sr No.	Paper	Approach	BER (Propagation channel NLOS/LOS)	BER (Modulation and coding)	Comparison with proposed technique using modulation and coding	Comparison with proposed technique LOS/NLOS
1	Al-Barrak et al. (2017)	RS performance is increased using multi-path propagation in LOS and NLOS scenario	10^−5^	x	x	10^−5^, 10^−6^
2	Mergu (2016)	RS performance is analyzed using RS codes concatenated with convolutional codes over AWGN channel	10^−2^	Convolutional codes, 10^−2^	10^−5^	10^−5^
3	Tiwari, Hirwe & Dubey (2013)	Comparison analysis of LDPC and RS codes on multiple antennas using AWGN channel	10^−2^ to 10^−3^	BPSK, QPSK, LDPC, RS, 10^−2^ to 10^−3^	10^−5^	10^−5^
4	Indoonundon & Pawan Fowdur (2021)	To achieve ultra reliable low latency communication in 5G, a detailed analysis of coding schemes was conducted	x	BER lies between 10^−6^ to 10^−7^	10^−7^	x
5	Saleh & Hasson (2019)	Communication reliability was improved using different diversity schemes	x	BPSK, MRC outperforms other techniques	10^−5^	x
6	Hajiyat et al. (2019)	The reliability of VANET was evaluated using turbo, low density parity check code (LDPC), polar code, systematic convolutional codes (SCC), and non-systematic convolutional codes (NSCC) coding types	x	10^−7^	10^−6^	x
7	Hamarsheh et al. (2022)	MIMO-FFH-OFDM	x	10^−4^	10^−5^	x

**Figure 15:**
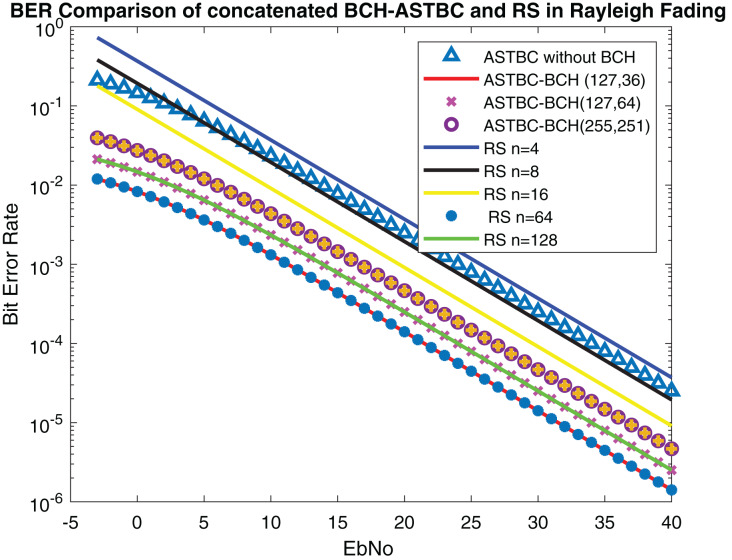
Comparison of BER of STBC-RS with BCH-ASTBC.

**Figure 16:**
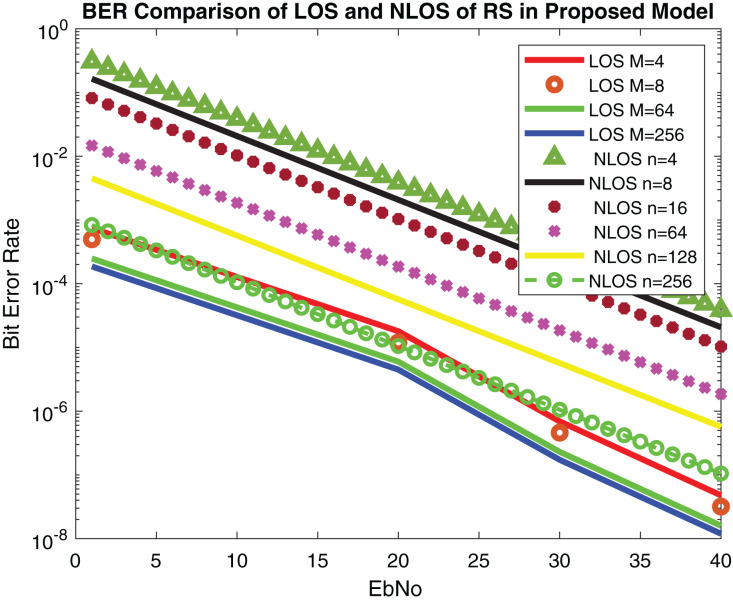
BER comparison of RS in Rayleigh fading and AWGN channel.

**Figure 17:**
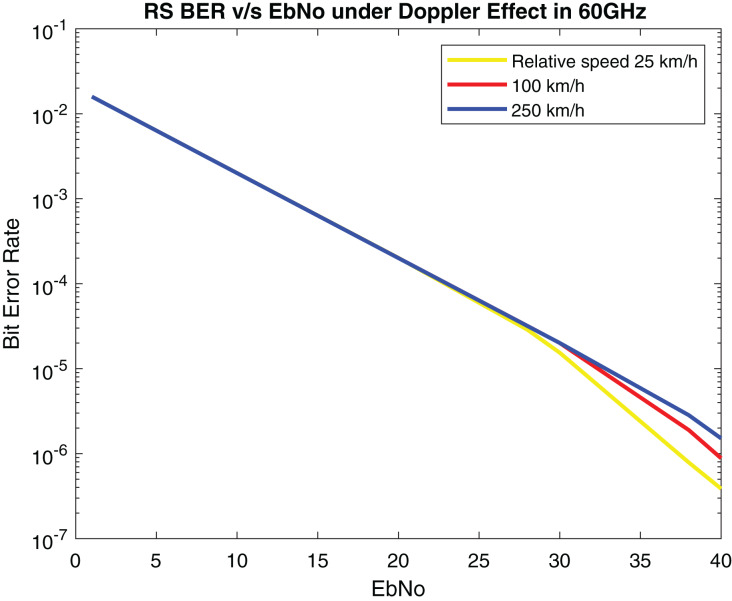
BER of RS under doppler effect.

The performance of the proposed system is compared with Ahmed, Rasheed & Liyanage (2021) in which concatenated BCH-ASTBC was used for VANET communication. Results are described in Fig. 15. The performance of BCH-ASTBC with code rate (12,736) is similar to RS BER at *n* = 64. The similar curve was also obtained for BCH-ASTBC with code rate (12,764) and RS BER at *n* = 128. The BER curve of ASTBC without BCH code is in between RS BER at *n* = 4 and RS BER at *n* = 8. Figure 16 depicts the comparative analysis of AWGN channel and Rayleigh channel. Figure 17 displays result of doppler shift in RS coding. The change in frequency causes frequency offset is the main source of high BER in VANET communications.

### Comparison analysis of proposed approach with IEEE 802.11bd and 3GPP V2X communication

**Figure 18:**
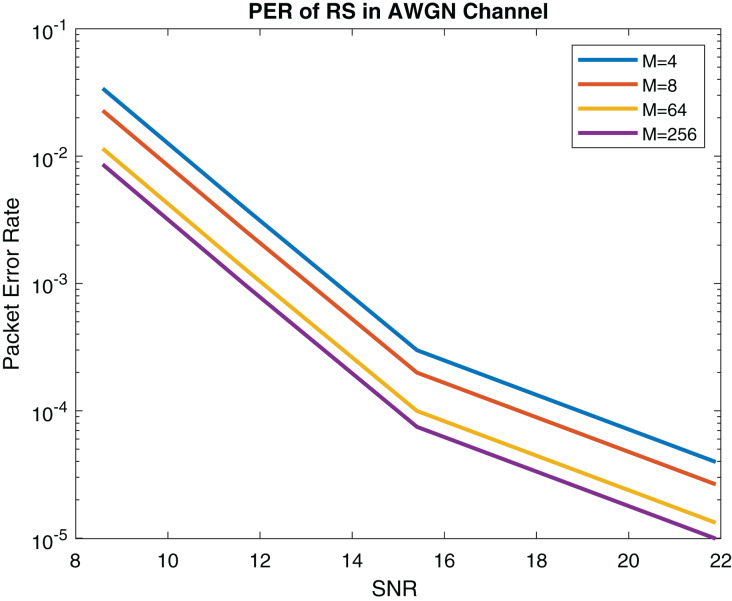
PER (Packet size = 100 Bytes) of RS in AWGN channel.

**Figure 19:**
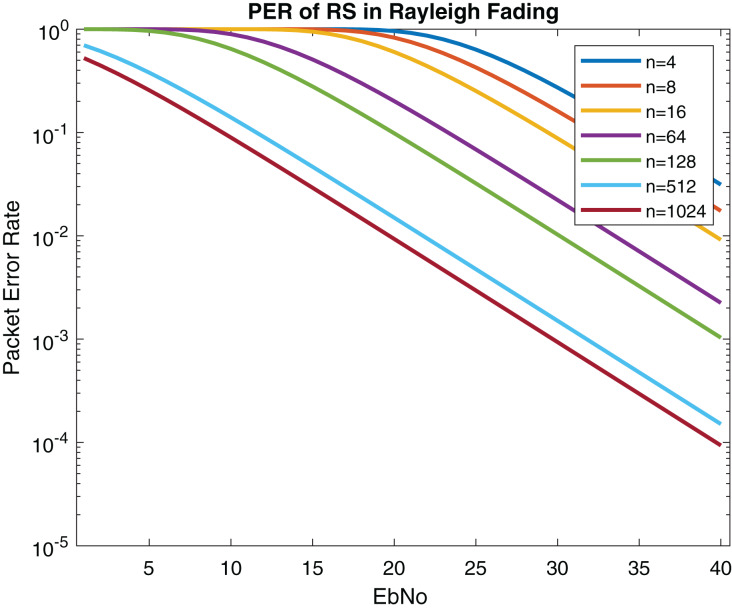
PER (Packet size = 100 Bytes) of RS in Rayleigh fading.

In this subsection, the performance of the proposed system is compared with IEEE 802.11bd and 5G NR V2X standard. The PER of LOS model is described in Fig. 18. The PER of NLOS model (Rayleigh fading) is shown in Fig. 19.

The performance of upcoming technologies *i.e*., 5G NRV2X and IEEE 802.11bd V2V communications was analyzed in Waqar, Norman & Gerhard (2019). Comparing RS packet error rate (PER) in AWGN channel shown in Fig. 18 with 802.11bd and NR V2X 64-QAM in Waqar, Norman & Gerhard (2019) it can be observed that the performance of our proposed RS model is optimal. Results are displayed in Table 4.

**Table 4:** PER analysis of proposed approach (Modulation) with IEEE 802.11p and NR V2X.

Comparison analysis of proposed approach with IEEE 802.11bd and V2X (Waqar, Norman & Gerhard, 2019)		
VANET communication standards	Modulation	PER
IEEE 802.11bd	QPSK/64QAM	10^−3^
NR V2X	QPSK/64QAM	10^−3^/10^−2^
Proposed methodology	BPSK/16/QAM/64QAM	10^−4^/10^−5^

According to Anwar et al. (2020) the performance of LDPC is marginal in V2X communication. The performance of proposed model outperforms LDPC, Turbo, Polar and Convolutional coding. Because the reliability of the system gets better since low PER is received in Fig. 19. Table 5 displays the comparison between proposed method with IEEE 802.11bd and V2X using PER. Comparing RS performance under Doppler Effect shown in Fig. 17, it can be remarked that RS performance is optimal.

**Table 5:** PER analysis of proposed approach (Coding) with IEEE 802.11p and NR V2X.

Comparison analysis of proposed approach with IEEE 802.11bd and V2X (Anwar et al., 2020)			
VANET communication standards	Modulation	Coding	PER
IEEE 802.11bd	BPSK	Convolutional, LDPC, Turbo, Polar	10^−3^
NR V2X	BPSK	Convolutional, LDPC, Turbo, Polar	10^−3^
Proposed methodology	BPSK	RS	10^−4^, 10^−5^

In Triwinarko, Dayoub & Cherkaoui (2021) the performance of various modulations schemes was evaluated on IEEE 802.11bd using LDPC. The packet size of 100 bytes was used for simulation. It can be observed that the RS error control coding gives us high reliability, as mentioned in Figs. 18 and 19.

Throughput of the proposed model is analyzed using equation below.

**Figure 20:**
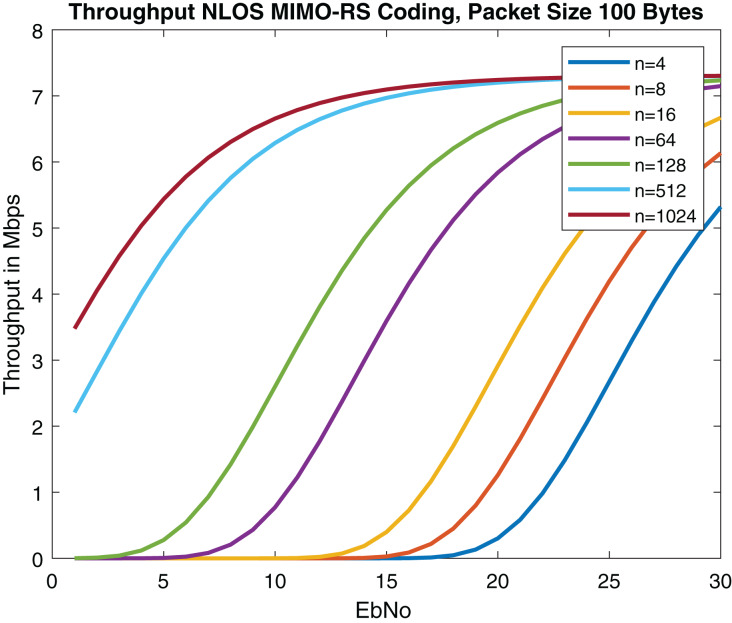
Throughput of MIMO-RS (Packet size = 100 Bytes) in NLOS.

$Throughput = R.(1 - PEP)$

where R corresponds to data rate. Results are described in Fig. 20. The results outperform with Anwar et al. (2020) and are comparable with Triwinarko, Dayoub & Cherkaoui (2021) as described in Table 6. 5G NR was integrated with Spatial Multiplexing MIMO while keeping other DSRC specifications the same (Dey et al., 2020). Convolution coding was used in the system. The performance of the 4 * 4 MIMO concatenated with 5G NR was analyzed using MMSE and ZF equalizers (Dey et al., 2020). We have used for 4 * 4 MIMO system. According to Obi et al. (2021), among maximum likelihood (ML) estimator zero forcing (ZF) and minimum mean square error (MMSE), ML performance is optimal.

**Table 6:** Throughput analysis of proposed approach with IEEE 802.11p and NR V2X.

Throughput analysis of proposed approach with IEEE 802.11bd and V2X (Anwar et al., 2020; Triwinarko, Dayoub & Cherkaoui, 2021)		
VANET communication standards	Coding	Throughput
IEEE 802.11bd	LDPC	7 Mbps
V2X	Convolutional, LDPC, Turbo, Polar	4 Mbps
Proposed methodology	RS	7 Mbps

**Figure 21:**
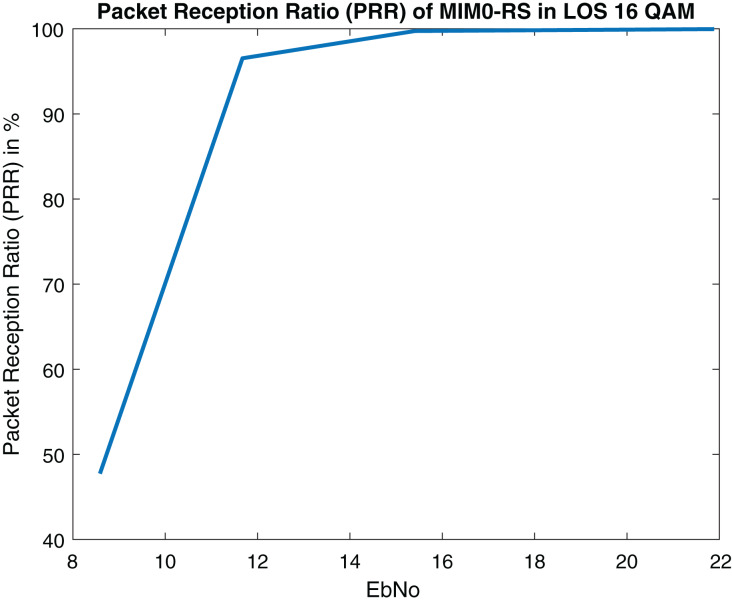
Packet reception ratio (PRR) of MIMO-RS in LOS (Packet size = 100 Bytes).

**Figure 22:**
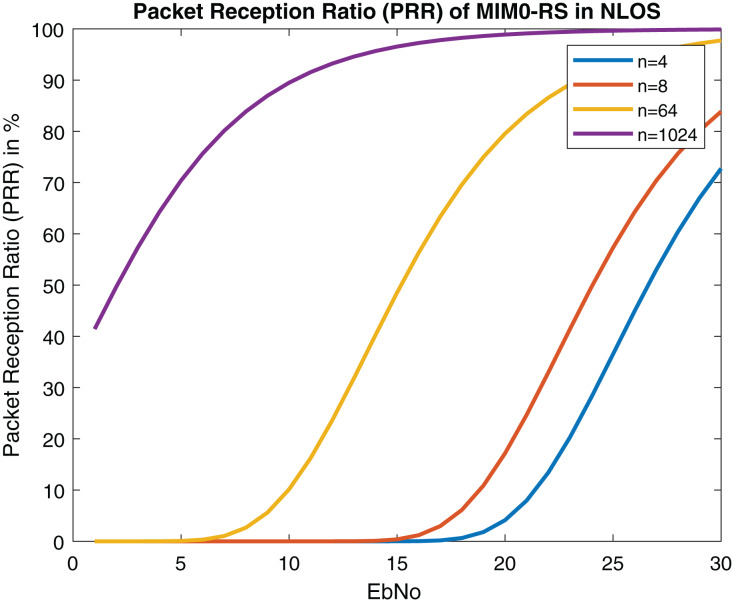
Packet reception ratio (PRR) of MIMO-RS in NLOS (Packet size = 100 Bytes).

Figures 21 and 22 depict PRR of both NLOS and LOS. Our proposed approach for channel modeling in VANET *i.e*., MIMO-STBC can be adopted in V2X communication and IEEE 802.11bd. Researchers are considering inducing MIMO-STBC in current VANET communication systems as mentioned above.

## Challenges and limitations of the proposed model

In the proposed model, multiple antennas and other hardware systems are used, so hardware complexity is higher. Since mathematical algorithms are used in the design of the beamforming system, a cutting-edge, highly processing DSP chip is required. Beamforming systems cost more than non-beamforming systems because they use more hardware resources and more sophisticated DSP chips. The use of more resources results in a higher power requirement for beamforming systems. Consequently, the beamforming system’s battery drains more quickly.

In some cases, perfect Channel State Information (CSI) is considered at the receiver. Reed Solomon codes work well for M-ary modulations schemes than BPSK systems.

## Conclusion

The future of VANET will be driven by mmWave communications. In this manuscript, a tractable model using STBC-RS is proposed for achieving ultra-reliability of 1- 10^−5^. The closed-form approximations of BER using RS in the AWGN channel and Rayleigh fading are derived. The results show that the proposed model outmatches previous BER estimation approaches of RS and STBC in 5G VANET networks. On comparing the model with existing VANET communicating systems it can be concluded that the proposed model performance is better than IEEE 802.11bd. We recommend that for designing V2X architectures, MIMO-STBC along with RS coding provides more useful results, since low PEP is received. The designed model can also be employed in 802.11p as a physical layer enhancement technique.

This research work provides the guidelines for quantitating the BER and PER in error control coding and a road map to design various VANET architectures. In future work, RS error probability can be analyzed for more number of antennas. Further, a VANET channel model for V2X communication can be developed using large antenna array sizes.

## Supplemental Information

10.7717/peerj-cs.1374/supp-1 Supplemental Information 1 MatLab codes.

## List of Abbreviations

ASE: Amplified Spontaneous Emission
ASTBC: Alamouti Space Time Block Coding
AT: Atmospheric Turbulence
BCH: Bose–Chaudhuri–Hocquenghem
C-ITS: Cooperative-Intelligent Transport System communications
FEC: Forward Error Correction
GSM: Global System Mobile
ICC: Interchannel Crosstalk
LOS: Line of Sight
MAC: Medium Access Control
MIMO: Multi-Input Multi-Output
NGV: Next-Generation V2X
NOMA: Non-Orthogonal Multiple Access
OFDM: Orthogonal Frequency Division Multiplexing
PHY: Physical Layer
PER: Packet Error Rate
PRR: Packet Reception Ratio
3GPP: 3rd Generation Partnership Project
16QAM: Quadrature Amplitude Modulation
RS: Reed Solomon
STBC: Space-Time-Block-Coding
SPM: Sub carrier-Power Modulation
VANET: Vehicular ad-Hoc Network
V2I: Vehicle to Infrastructure
V2X: Vehicle to Everything
V2V: Vehicle to Vehicle

## List of Notations and Definitions

H_ef_^H^: conjugate transpose of H_ef_
$km/h$: Kilo meter per hour
$\gamma$: Signal-to -Noise Ratio
$\psi$: far-zone phase difference between adjacent elements
$\theta$: represents angle of arrival in beamforming
$\phi$: represents angle of reflection
s: corresponds to periodicity of complex weight
N: transmitting antennas
d: equidistant spacing between elements
${f_d}$: Doppler shift
v: Relative velocity
n: Coded bits

## Additional Information and Declarations

### Data Availability

The following information was supplied regarding data availability: The code is available in the Supplemental Files.
